# Job vacancy: Eurosurveillance is looking for a Scientific Editor

**DOI:** 10.2807/1560-7917.ES.2016.21.44.30385

**Published:** 2016-11-03

**Authors:** Eurosurveillance editorial team

**Affiliations:** 1 European Centre for Disease Prevention and Control (ECDC), Stockholm, Sweden

The European Centre for Disease Prevention and Control (ECDC) invites applications for the following position:

• Scientific Editor Eurosurveillance (RMC)

The deadline is 28 November 2016. For more information, visit the ECDC website: http://ecdc.europa.eu/en/aboutus/jobs/Pages/JobOpportunities.aspx

